# Red blood cell distribution width and outcome in trauma patients

**DOI:** 10.1515/jom-2020-0089

**Published:** 2021-02-01

**Authors:** McKenzie Brown, Sean Nassoiy, Timothy Plackett, Fred Luchette, Joseph Posluszny

**Affiliations:** Department of Surgery and Public Health Sciences, Loyola University Medical Center, Maywood, IL, USA; Department of Surgery and Public Health Sciences, Loyola University Medical Center, Maywood, IL, USA; Department of Surgery and Public Health Sciences, Loyola University Medical Center, Maywood, IL, USA; and 759th Forward Surgical Team, BLDG A-6631 Gorham Street, 28310-0001 Fort Bragg, NC, USA; Department of Surgery and Public Health Sciences, Loyola University Medical Center, Maywood, IL, USA; Department of Surgery, Edward Hines Jr. Veterans Administration Hospital, Hines, IL, USA; Department of Surgery and Public Health Sciences, Loyola University Medical Center, Maywood, IL, USA

**Keywords:** mortality, red blood cells, red blood cell distribution width, sepsis, trauma

## Abstract

**Context:**

Red blood cell distribution width (RDW) has been used to predict mortality during infection and inflammatory diseases. It also been purported to be predictive of mortality following traumatic injury.

**Objective:**

To identify the role of RDW in predicting mortality in trauma patients. We also sought to identify the role of RDW in predicting the development of sepsis in trauma patients.

**Methods:**

A retrospective observational study was performed of the medical records for all adult trauma patients admitted to Loyola University Medical Center from 2007 to 2014. Patients admitted for fewer than four days were excluded. Admission, peak, and change from admission to peak (Δ) RDW were recorded to determine the relationship with in-hospital mortality. Patient age, development of sepsis during the hospitalization, admission to the intensive care unit (ICU), and discharge disposition were also examined.

**Results:**

A total of 9,845 patients were admitted to the trauma service between 2007 and 2014, and a total of 2,512 (25.5%) patients fit the inclusion criteria and had both admission and peak values available. One-hundred twenty (4.6%) died while in the hospital. RDW values for all patients were (mean [standard deviation, SD]): admission 14.09 (1.88), peak 15.09 (2.34), and Δ RDW 1.00 (1.44). Admission, peak, and Δ RDW were not significant predictors of mortality (all p>0.50; hazard ratio [HR], 1.01–1.03). However, trauma patients who eventually developed sepsis had significantly higher RDW values (admission RDW: 14.27 (2.02) sepsis vs. 13.98 (1.73) no sepsis, p<0.001; peak RDW: 15.95 (2.55) vs. 14.51 (1.97), p<0.001; Δ RDW: 1.68 (1.77) vs. 0.53 (0.91), p<0.001).

**Conclusion:**

Admission, peak, and Δ RDW were not associated with in-hospital mortality in adult trauma patients with a length of stay (LOS) ≥four days. However, the development of sepsis in trauma patients is closely linked to increased RDW values and in-hospital mortality.

Red blood cell distribution width (RDW) is a quantitative measure of variation in the size of circulating red blood cells (RBCs).^1^ RDW is a component of the routine complete blood count. It is calculated by dividing the standard deviation of the mean RBC size by the mean corpuscular volume of the RBCs and multiplying by 100 to convert this number to a percentage. The normal range for RDW is 11.0–15.0%.^1^ Traditionally, RDW helps differentiate types of anemia; however, more recently, RDW has been used to predict mortality in a wide range of clinical manifestations including necrotizing fasciitis, pancreatitis, and sepsis.^2–5^

The role of RDW in predicting mortality in trauma patients has been studied but remains unclear. In one of the first studies on this subject, Majercik et al.^6^ evaluated the association between admission RDW and 30-day and one-year mortality in trauma patients. After dividing patients into quintiles based on admission RDW, the upper quintile (i.e., highest RDW) had the highest mortality. These findings were further supported by Kong et al.,^7^ who found that RDW values at one and two days after injury were predictive of 28-day mortality. However, an association between RDW and mortality from trauma is not without equipoise. Paulusetal.^8^ evaluated the association between admission RDW and the need for massive transfusion. Although admission RDW independently predicted massive transfusion, there was no association between admission RDW and mortality in a multivariate analysis. Likewise, Habibpour et al.^9^ found no association between the RDW on admission or 24 hours after admission and mortality. Differences in sample size may explain the disparate outcomes observed in these studies.

Given the prognostic value of RDW values in multiple disease processes, the conflicting and limited data in trauma, and the ease of availability of RDW values, the objective of this study was to determine whether admission, peak, and change from admission to peak (Δ) RDW can predict mortality in trauma patients. Secondary endpoints of admission to the intensive care unit (ICU), developing sepsis during the hospitalization, and discharge disposition were also examined. These secondary endpoints were chosen based on previous studies in which other disease processes demonstrated a relationship between RDW and these endpoints.^5,10^

When considering mortality following trauma, sepsis is a particularly salient secondary endpoint. Deaths from trauma are traditionally thought of as following either a trimodal or, more recently, bimodal distribution in which early deaths are attributed to hemorrhage and the immediate sequala of injury and late deaths are from sepsis and multiorgan failure.^11^ As such, earlier identification of patients with a higher risk of developing sepsis may allow an opportunity to initiate interventions that stave off this complication. Considering the relationship between RDW and inflammation,^1^ we hypothesized that a high or rising RDW may be a marker for developing sepsis and therefore could function as an early identifier of at-risk patients.

## Methods

After approval was obtained for this study from Loyola University Medical Center’s Institutional Review Board, a retrospective chart review was performed. The Loyola University Medical Center’s admissions clinical database was queried to identify all patients admitted to the trauma surgery service from 2007 to 2014. All adults (≥18 years old) with a length of stay (LOS) ≥four days were included. Patients with shorter LOSs were excluded to eliminate patients with minor injuries and immediate deaths. After the patients of interest were identified, individual patient charts were retrospectively reviewed, and the following information was abstracted: age, sex, ethnicity, ICU admission, development of sepsis, in-hospital mortality, and discharge disposition (home, skilled nursing facility [SNF], long-term acute care [LTAC], hospital transfer). The RDW at the time of admission, the highest (peak) RDW during the hospitalization, and the Δ RDW were also recorded during the review of the individual patient records. The primary outcome was in-hospital mortality. The secondary outcomes were the relationship of RDW with ICU status, the development of sepsis, and discharge disposition.

The attending physician’s clinical diagnoses in the medical record were used to identify whether a patient had sepsis. More specifically, if a patient had any of the following ICD-9 codes documented, he or she was considered to have developed sepsis: 020.0, 038.XX 112.5, 112.81, 117.9, 276.2, 286.6, 286.9, 287.3–5, 293, 348.1, 348.3, 458, 570, 518.81, 518.82, 518.85, 572.2, 572.3, 580, 584, 780.01, 780.09, 785.5, 785.52, 790.7, 799.1, 995.91, 995.92.^12–14^ The veracity of the sepsis diagnosis was not independently verified through review of culture results, physiologic variables, and/or laboratory tests. The standard clinical practice of the trauma service at that time was to utilize the Sepsis-2 criteria to diagnose sepsis (i.e., meeting the criteria for the systemic inflammatory response syndrome and having a positive culture result).^15^

Statistical analysis was performed using SAS version 9.4 (SAS Institute), and statistical significance was attributed to p<0.05. A t-test was used to compare mean RDW value (admission, peak, and Δ RDW) and patient demographics and clinical outcomes. Post-hoc tests were performed to control for type I error. A Satterthwaite correction was used for mortality, ICU, and sepsis status to estimate the effective degrees of freedom. A Welch correction was used for discharge disposition.

Mortality as a function of RDW value (admission, peak, and Δ RDW) was evaluated with a Cox proportional hazard model. A proportional hazard ratio (HR) was used to describe the chance of an event occurring between the event of interest (mortality) and each incremental increase in the RDW or Δ RDW. An HR of 1.0 indicated no relationship between mortality and increasing RDW or Δ RDW, whereas a value >1.0 indicated increased mortality as the RDW or Δ RDW rises. The proportional hazards assumption was evaluated as described by Lin et al.^16^ After initially determining HRs for mean RDW values for admission, peak, and Δ RDW, a second analysis was performed using a binary cutoff value of < or ≥17. RDW values were also divided into quintiles, and a Kaplan–Meier (KM) time-to-event analysis was performed to assess the cumulative incidence of all-cause mortality as a function of each quintile. Mortality HRs were also calculated based upon age, admission to the ICU, and sepsis to examine factors other than RDW that may be associated with mortality.

A secondary analysis was repeated using sepsis as the studied outcome. A t-test was used to compare mean RDW value (admission, peak, and Δ RDW) and the development of sepsis during the hospital stay. A post hoc Satterthwaite correction was also performed. A Wilcoxon rank-sum test was used to test LOS and ICU LOS on sepsis. Sepsis was then evaluated as a function of multivariable LOS and Δ RDW. A multivariable binary logistic regression model was used to assess the odds of sepsis given Δ RDW and LOS. Given the close relationship between sepsis and Δ RDW, a multivariable binary logistic regression model was then used to calculate HRs and 95% confidence intervals (CIs) to assess survival as a function of Δ RDW and sepsis.

## Results

There were 9,845 patients admitted to the trauma service between 2007 and 2014, including admissions from the emergency room as well as patients transferred from outside hospitals. A total of 6,970 patients were excluded for LOS ≤three days or age <18 years. An additional 363 patients were excluded due to incomplete laboratory results and multiple admissions. For the remaining 2,512 patients (25.5% of all admitted trauma patients), the average age was 49 ± 20 years; 1,719 (68.4%) were men and 792 (31.5%) were women. A majority (2,145; 85.4%) were admitted to the ICU, and 1,131 (45%) developed sepsis during their hospital stay. The average LOS was nine days (range, four to 128 days). In-hospital mortality was 4.7% (n=120). In terms of discharge disposition, 1,425 (56.7%) were discharged to home, 819 (32.6%) were discharged to an SNF, 89 (3.5%) were discharged to an LTAC, and 63 (2.5%) were transferred to another hospital (Table 1). Mean (SD) RDW values for all patients were: admission 14.09 ± 1.88, peak 15.09 ± 2.34, and Δ RDW 1.00 ± 1.44.

**Figure 1:**
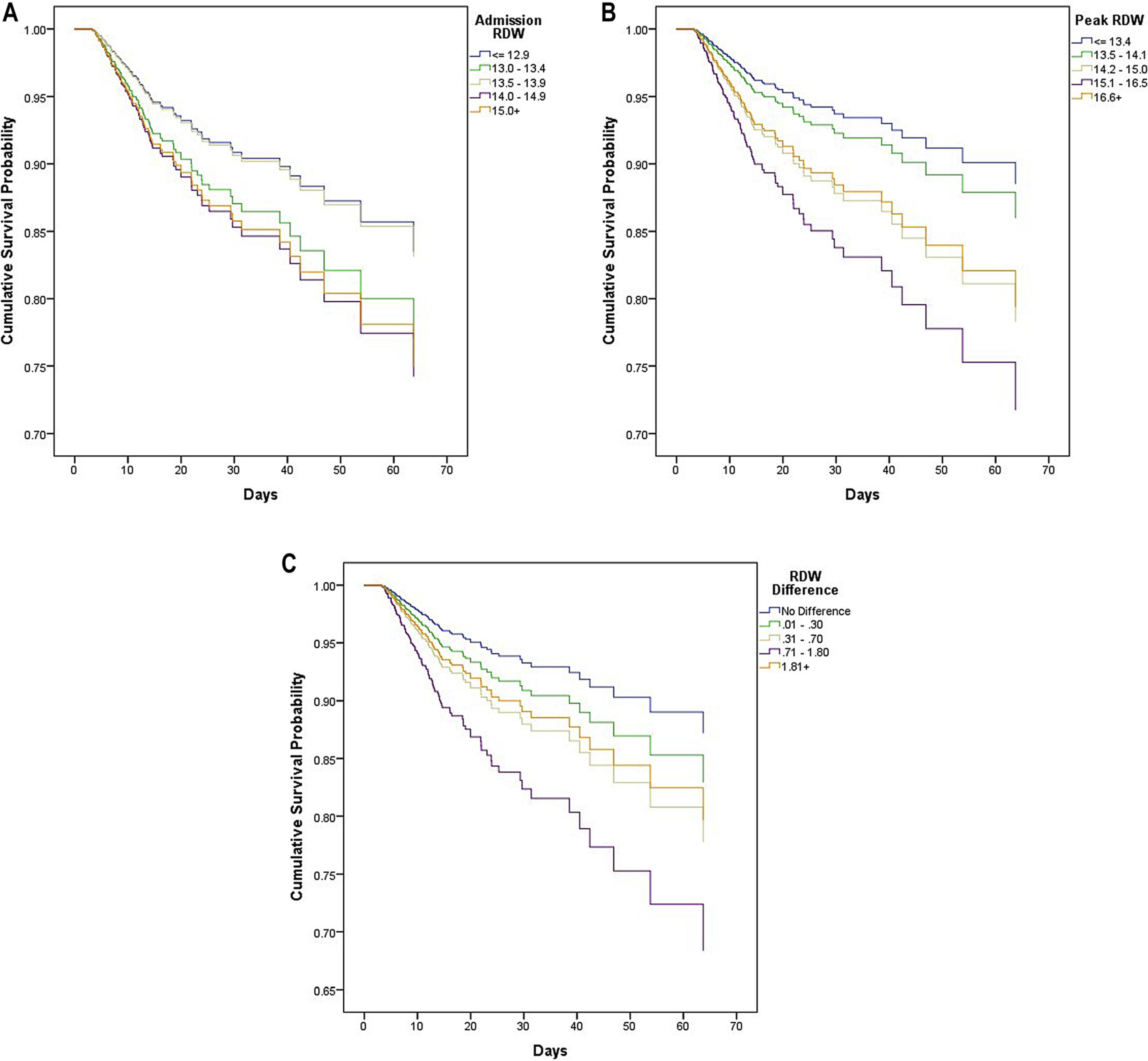
(A) Kaplan Meier survival curve, admission red blood cell distribution width (RDW). (B) Kaplan Meier survival curve, peak RDW. (C) Kaplan Meier survival curve, Δ RDW.

Using a Cox proportional hazards model, admission (HR=1.03 [0.95–1.12]), peak (HR=1.02 [0.96–1.10]), and Δ RDW (HR=1.01 [0.91–1.12]) were not significant predictors of mortality (p=0.50, 0.50, and 0.86, respectively; Table 2a). When admission and peak RDW values were collapsed into binary values of < or ≥17 to assess the significance of the highest RDW values, admission (HR=0.87 [0.47–1.63] and 1.14 [0.61–2.13], respectively) and peak RDW (HR=1.20 [0.79–1.82] and 0.84 [0.55–1.27], respectively) were not significant predictors of mortality (p=0.67 and 0.40, respectively; Table 2b). Admission, peak, and Δ RDW values were then grouped into quintiles. In doing so, only the fourth highest quintile for both peak (HR=2.72 [1.14–6.51]) and Δ RDW (HR=2.78 [1.24–6.26]) were statistically significant for mortality. Thus, only a peak RDW ≥16.68 and Δ RDW ≥1.81 were associated with a significantly increased risk of mortality on a Cox proportional hazard model. When these data were expressed in a KM curve, the overall model for Δ RDW was statistically significant with a p-value of0.04; however, neither admission nor peak RDW models were statistically significant (Figure 1A–C).

Independent factors associated with mortality included age (HR=1.03 [1.02–1.04]), admission to the ICU (HR=7.30 [1.01–52.55]), and the development of sepsis (HR=4.58[2.49–8.41]). Patients with sepsis had significantly higher RDW values (admission, 14.27 ± 2.02 sepsis vs. 13.98 ± 1.73 no sepsis, p<0.001; peak, 15.95 ± 2.55 vs. 14.51 ± 1.97, p<0.001; Δ RDW, 1.68 ± 1.77 vs. 0.53 ± 0.91, p<0.001) and longer duration of hospital stay (median, 13.26 days [7.5–22.53] vs. 6.30 [4.69–9.56]; Table 3). Among those patients admitted to the ICU, patients with sepsis also had a significantly longer hospital stay than those without sepsis (median, nine days [four to 18] vs. three [two to five]). After holding LOS constant, a one-unit increase in the Δ RDW value increased the odds of sepsis by 52% (OR=1.52 [1.38–1.67]; p<0.001). Similarly, after holding Δ RDW constant, a one-day increase in LOS was expected to increase the odds of sepsis by 10% (OR=1.10 [1.08–1.12]; p<0.001; Table 4).

The closest association was found between sepsis and Δ RDW. Therefore, we tested mortality as a function of both Δ RDW and the development of sepsis. Independently, Δ RDW was not associated with mortality (HR=0.94 [0.84–1.04]; p=0.23). However, the development of sepsis was associated with mortality independent of Δ RDW (HR=4.87 [2.62–9.03]; p<0.001; Table 5).

## Discussion

In a large cohort of trauma patients admitted to a Level I trauma center, admission, peak, and Δ RDW were found to not correlate with mortality. However, consistent with the current RDW literature, admission, peak, and Δ RDW did correlate with the eventual development of sepsis after injury.

Although the association between RDW and mortality has been well documented in many medical conditions,^2–5^ there has been limited information regarding its role in the trauma population. Four groups have previously attempted to address this issue, with divergent results. Majercik et al.^6^ demonstrated that admission RDW independently predicted sex-specific mortality in trauma patients at 30 days and one year. Kong et al.^7^ demonstrated that RDW values at one and two days after admission predicted morality in trauma patients at 28 days. However, the small sample size raises concerns for a type I error. Paulus et al.^8^ reported that their data ‘corroborated’ the findings by Majercik’s group; however, they were unable to demonstrate statistical significance and should be viewed as rejecting the conclusion of Majercik’s group.^6^ Habibpour et al.^9^ also found no significant relationship between RDW on admission or one day after injury and mortality. However, as with Kong et al.,^7^ this study was too small to reach a definitive conclusion. In fact, much of the equipoise from these four prior studies can be attributed to a difference in sample size and the risk for committing a type I error. Within this context, the previous studies seem to suggest that the admission RDW may not be an independent marker for mortality following traumatic injury.

In the present study, our data showed a lack of correlation between RDW and mortality that held true when RDW was evaluated as both a univariable value and with binary cut score values (< or ≥17). RDW was also divided into quintiles, similar to prior studies, and although the overall Δ RDW model was statistically significant, only the fourth quintile appeared distinct when this data were expressed as a KM curve. If elevated RDW values were truly predictive of mortality, there would be a graded increase in mortality as RDW values increased from the first to the fourth quintile. This trend was not demonstrated among our admission, peak, or Δ RDW models. When combined with the previous studies, we believe that the current literature supports a conclusion that RDW is not an independent predictor of mortality following trauma. However, acknowledging the limitations of the studies, we believe there is a role for ongoing research.

Although we did not find a correlation between RDW and mortality, we considered a secondary endpoint of sepsis. In both the bimodal and trimodal models of mortality from trauma, sepsis is the predominant cause of late deaths.^11^ Given that most previous studies of RDW and critically ill patients have demonstrated a clear relationship between higher RDW values and sepsis,^5,10,17^ we sought to demonstrate whether such a relationship held true for trauma patients. The present study demonstrates that Δ RDW correlated with sepsis because an elevated Δ RDW was associated with a nearly fivefold increase in the incidence of sepsis.

The link between RDW and sepsis for trauma patients is in keeping with the association between RDW and inflammation and infection.^18,19^ In fact, RDW may be more predictive of outcome in patients with infectious or inflammatory diagnoses rather than noninfectious diagnoses.^18^ Further research is needed to understand the relationship between the inflammation associated with traumatic injury, the presence of an infectious complication after injury, and how these two events interact with the RDW.

It is our belief that admission RDW is more reflective of the patient’s baseline comorbidities and less an indication of the severity of their injuries. To reflect more of the effect of the trauma rather than baseline health status, we uniquely evaluated both peak and Δ RDW values. Although we did not find that peak or Δ RDW were associated with mortality, further analysis did reveal a significant association between peak and Δ RDW values and the development of sepsis in trauma patients, and this association was greatest with Δ RDW. The association between elevated RDW and sepsis has already been established in several studies.^20,21^ Within this patient population, it has been demonstrated that individuals with the highest RDW values have an associated increased risk of death. Our study is the first we know of to demonstrate that an elevated RDW may be clinically useful as a marker for sepsis in trauma patients. Given the close relationship between RDW and the development of sepsis,^22^ further studies should focus on utilizing RDW as a marker of the development, persistence, and possible resolution of sepsis in trauma patients.

## Limitations

Our study had several limitations. This study was a retrospective observational study of an administrative database and electronic health record. Due to a limitation of the database and health record, we were unable to include severity of illness markers, such as injury severity score (ISS) or Apache, which prevented performing propensity matching. We also used ICD-9 codes and admission services to identify patients of interest, which allows for selection bias. In addition, our use of an admission database did not allow us to distinguish between patients admitted through the ED and those patients transferred from an outside hospital, which could have altered admission RDW values.

A larger cohort of patients may help to better delineate some of our results reported in the KM curve that suggested that higher quintiles of peak RDW were associated with mortality. However, the study population would need to be three times larger in order to include enough of these higher quintile patients to achieve adequate power. A multi-institutional study would be better suited for such a study and would increase the generalizability of the results.

Finally, it was unknown which patients received a transfusion at admission and the role that this could potentially have on altering RDW values. In keeping with the literature that preceded this study,^1–7^ the potential effect of transfusion on RDW was not taken into consideration. Intuitively, the transfusion of RBCs would seem likely to alter the patient’s RDW following their administration and thereby affect the peak RDW and Δ RDW values. A recent pilot study supports the hypothesis that the RDW is affected by the transfusion of packed RBCs.^23^ However, how transfusion influences the relationship between RDW and outcomes is only just beginning to be investigated, and early studies show differing results.^24,25^ Given that it is plausible for peak RDW and Δ RDW to be confounded by transfusions, any future studies must strive to account for this possibility.

Whether or not interventions can decrease the RDW and thereby affect outcomes remains speculative and is beyond the reach of this study. However, it is tempting to wonder whether interventions that have been shown to decrease inflammation, such as osteopathic manipulative therapy,^26^ could lead to an improved RDW and improved outcomes. Future study into this possibility is warranted. However, for now, the practical use of this data is for guiding discussion with the patient and their family on expected outcomes. This common laboratory finding provides additional objective data that can be used when answering questions about the anticipated LOS, the need for ICU admission, and the risk of getting sicker (i.e., developing sepsis) before fully recovering.

## Conclusion

In conclusion, admission, peak, and Δ RDW values were not associated with in-hospital mortality in adult trauma patients with LOS ≥four days. In contrast, the development of sepsis in trauma patients was closely linked to higher RDW values and in-hospital mortality. Consistent with the literature, our study showed that higher RDW values were associated with inflammation (sepsis) and higher mortality in this group. Better understanding of the relationship between RDW and inflammation/sepsis may help in determining how RDW may be used to recognize the onset and persistence of an inflammatory state and its relationship to mortality in trauma.

## Figures and Tables

**Table 1:** Change in RDW as a function of univariable demographics.

	n=2,512*	Mean (SD) RDW					
		Admission	p-value	Peak	p-value	δ	p-value
**Sex**	2,511		<0.001		<0.001		0.17
Female	792	14.61 (2.24)		15.71 (2.66)		1.10 (1.52)	
Male	1,719	13.87 (1.61)		14.89 (2.15)		1.02 (1.45)	
**Race**	2,510		<0.001		0.01		0.21
White	1,575	14.12 (1.84)		15.20 (2.30)		1.08 (1.44)	
Black	504	14.31 (1.96)		15.26 (2.39)		0.95 (1.43)	
Other	431	13.81 (1.84)		14.84 (2.50)		1.02 (1.62)	
**Ethnicity**	2,495		<0.001		<0.001		0.57
Hispanic	436	13.70 (1.57)		14.78 (2.21)		1.08 (1.54)	
Non-hispanic	2,059	14.19 (1.91)		15.23 (2.38)		1.04 (1.46)	
**Disposition**	2,512		<0.001		<0.001		<0.001
Home	1,425	13.95 (1.77)		14.64 (2.07)		0.70 (1.11)	
Deceased	116	14.38 (1.67)		16.35 (2.34)		1.96 (1.85)	
Skilled nursing facility	819	14.36 (2.07)		15.75 (2.60)		1.39 (1.71)	
Hospital transfer	63	13.71 (1.23)		14.53 (1.65)		0.82 (1.10)	
Long-term acute care	89	14.31 (1.77)		16.68 (2.25)		2.37 (1.90)	
**Hospital mortality**	2,512		0.10		<0.001		<0.001
Alive	2,396	14.09 (1.88)		15.09 (2.34)		1.00 (1.44)	
Deceased	116	14.38 (1.67)		16.35 (2.34)		1.96 (1.85)	
**ICU status**	2,512		<0.001		<0.001		<0.001
No	386	13.77 (1.42)		13.97 (1.49)		0.20 (0.38)	
Yes	2,126	14.17 (1.93)		15.36 (2.42)		1.20 (1.54)	
**Sepsis status**	2,512		<0.001		<0.001		<0.001
No	1,392	13.98 (1.73)		14.51 (1.97)		0.53 (0.91)	
Yes	1,120	14.27 (2.02)		15.95 (2.55)		1.68 (1.77)	
**Age, years**	2,512		<0.001		<0.001		0.20
18–40	970	13.52 (1.26)		14.52 (1.97)		1.00 (1.50)	
41–64	918	14.21 (1.94)		15.24 (2.35)		1.03 (1.42)	
≥65	624	14.87 (2.21)		16.00 (2.62)		1.13 (1.52)	

* n=2,512/2,545 (99%) of the sample had both admission and peak RDW values available. ICU, intensive care unit; SD, standard deviation; RDW, red blood cell distribution width.

**Table 2a:** Survival as a function of univariable RDW values (n=2512).

Mean (SD) RDW	Living	Deceased	Hazard ratio	p-value*
Admission	14.09 (1.88)	14.38 (1.67)	1.03 (0.95–1.12)	0.50
Peak	15.09 (2.34)	16.35 (2.34)	1.02 (0.96–1.10)	0.50
Difference	1.00 (1.44)	1.96 (1.85)	1.01 (0.91–1.12)	0.86

* Significance (p) was determined using the Cox proportional hazards model with observed proportional hazards. RDW, red blood cell distribution width; SD, standard deviation from the mean.

**Table 2b:** Survival as a function of univariable binary RDW values (n=2512).

	Living	Deceased	Hazard ratio	p-value*
**Admission**				0.67
**RDW**				
<17	2,236 (93.3%)	105 (90.5%)	0.87 (0.47–1.63)	
≥17	160 (6.7%)	11 (9.5%)	1.14 (0.61–2.13)	
**Peak RDW**				0.40
<17	2,001 (83.5%)	83 (71.6%)	1.20 (0.79–1.82)	
≥17	395 (16.5%)	33 (28.4%)	0.84 (0.55–1.27)	

* Significance (p) was determined using the Cox proportional hazards model with observed proportional hazards. RDW, red blood cell distribution width.

**Table 3:** Sepsis as a function of univariable RDW, LOS, and ICU LOS (n=2545).

	n	Sepsis status		p-value
		No (n=1,414)	Yes (n=1,131)	
Admission, mean (SD)	2,512	13.98 (1.73)	14.27 (2.02)	<0.001
Peak, mean (SD)	2,512	14.51 (1.97)	15.95 (2.55)	<0.001
Difference, mean (SD)	2,512	0.53 (0.91)	1.68 (1.77)	<0.001
**Median LOS in days, IQR**	2,545	6.30 (4.69–9.56)	13.26 (7.50–22.53)	<0.001
**Median ICU-LOS in days, IQR**	2,145	3.00 (2.00–5.00)	9.00 (4.00–18.00)	<0.001

ICU, intensive care unit (n=2,145; IQR, interquartile range; LOS, length of stay; RDW, red blood cell distribution width; SD, standard deviation.

**Table 4:** Sepsis as a function of multivariable LOS and RDW difference score (n=2512 of 2545).

	Odds ratio (95% CI)	p-value*
Difference from admission to peak RDW	1.52 (1.38–1.67)	<0.001
LOS in days	1.10 (1.08–1.12)	<0.001

* Significance (p) is based on a multivariable binary logistic regression model. Overall model χ^2^ (2)=650.36, p<0.001. CI, confidence interval; LOS, length of stay; RDW, red blood cell distribution width.

**Table 5:** Survival as a function of RDW difference score and sepsis (n=2512 of 2545).

	Hazard ratio (95% CI)	p-value*
Difference from admission to peak RDW	0.94 (0.84–1.04)	0.23
Sepsis	4.87 (2.62–9.03)	<0.001

* Significance (p) is based on a multivariable binary logistic regression model. Overall model χ^2^(2)=29.70, p<0.001.The interaction of RDW difference and sepsis was not significant (p=0.097). CI, confidence interval; LOS, length of stay; RDW, red blood cell distribution width.
